# Insights into the Trypanosome-Host Interactions Revealed through Transcriptomic Analysis of Parasitized Tsetse Fly Salivary Glands

**DOI:** 10.1371/journal.pntd.0002649

**Published:** 2014-04-24

**Authors:** Erich Loza Telleria, Joshua B. Benoit, Xin Zhao, Amy F. Savage, Sandesh Regmi, Thiago Luiz Alves e Silva, Michelle O'Neill, Serap Aksoy

**Affiliations:** 1 Yale School of Public Health, Department of Epidemiology of Microbial Diseases, LEPH, New Haven, Connecticut, United States of America; Fundaçao Oswaldo Cruz, Brazil

## Abstract

The agents of sleeping sickness disease, *Trypanosoma brucei* complex parasites, are transmitted to mammalian hosts through the bite of an infected tsetse. Information on tsetse-trypanosome interactions in the salivary gland (SG) tissue, and on mammalian infective metacyclic (MC) parasites present in the SG, is sparse. We performed RNA-seq analyses from uninfected and *T. b. brucei* infected SGs of *Glossina morsitans morsitans*. Comparison of the SG transcriptomes to a whole body fly transcriptome revealed that only 2.7% of the contigs are differentially expressed during SG infection, and that only 263 contigs (0.6%) are preferentially expressed in the SGs (SG-enriched). The expression of only 37 contigs (0.08%) and 27 SG-enriched contigs (10%) were suppressed in infected SG. These suppressed contigs accounted for over 55% of the SG transcriptome, and included the most abundant putative secreted proteins with anti-hemostatic functions present in saliva. In contrast, expression of putative host proteins associated with immunity, stress, cell division and tissue remodeling were enriched in infected SG suggesting that parasite infections induce host immune and stress response(s) that likely results in tissue renewal. We also performed RNA-seq analysis from mouse blood infected with the same parasite strain, and compared the transcriptome of bloodstream form (BSF) cells with that of parasites obtained from the infected SG. Over 30% of parasite transcripts are differentially regulated between the two stages, and reflect parasite adaptations to varying host nutritional and immune ecology. These differences are associated with the switch from an amino acid based metabolism in the SG to one based on glucose utilization in the blood, and with surface coat modifications that enable parasite survival in the different hosts. This study provides a foundation on the molecular aspects of the trypanosome dialogue with its tsetse and mammalian hosts, necessary for future functional investigations.

## Author Summary

Tsetse flies transmit the causative agents of African sleeping sickness and nagana in sub-Saharan Africa. The parasites are acquired when tsetse flies feed on an infected host, undergo multiplication in the fly gut and migrate to the salivary glands (SG). The cycle resumes once this infected fly transmits the parasites in conjunction with saliva to another host when feeding. We compared gene expression changes between parasitized and uninfected tsetse SG. We also assessed changes in parasite gene expression in the tsetse SG in relation to those present within vertebrate blood. We found that parasite infections increase expression of host proteins associated with stress and cell division, indicative of extensive cellular damage in SG. We also found that parasite infections reduce expression of the most highly expressed SG-specific secreted proteins, suggesting modification of saliva composition. The parasite transcriptome reveals changes in specific cell surface proteins and in metabolism related to glucose-amino acid utilization in the different host environments. This study provides information for critical understanding of tsetse-trypanosome interactions, and transcriptional changes that likely enable the parasite to persist in the varying environment of its insect and vertebrate hosts.

## Introduction

African trypanosomes are transmitted to the mammalian host through the bite of infected tsetse flies (*Glossina* spp.) [1]. Human African trypanosomiasis (HAT) is caused by *Trypanosoma brucei rhodesiense* and *Trypanosoma brucei gambiense*, while African animal trypanosomiasis (AAT) is caused by *Trypanosoma brucei brucei*, *Trypanosoma congolense* and *Trypanosoma vivax* [2]. To control disease in the mammalian host, no vaccines are available, and drugs used for treatment are expensive and have adverse side effects [3], [4], [5]. Progress has been made through methods that reduce tsetse population, but such programs remain expensive and re-infestation frequently occurs when they are abandoned [2]. A better understanding of the biological processes that underlie parasite transmission dynamics can lead to novel molecules, or methods for disease control. Multiple points of intervention, such as interference with the parasite's development in the insect host, or with its transmission from the insect to the mammalian host, or targeting the development of the parasite in the mammalian host in the early stages of infection can block disease spread. Studies in humans that aim to reduce malaria have targeted *Plasmodium* spp. antigens expressed in the sporozoite stages of the parasite present in mosquito saliva for the development of pre-erythrocytic malaria vaccines [6], [7].

Many studies have addressed the biology of trypanosomes in the mammalian host [8], but there is sparse information on the parasite differentiation and development processes in the tsetse host. Information on host-parasites interactions in the SG, the process of metacyclogenesis (development of mammalian infective parasites in the SG), and the establishment of mammalian infections upon transmission in fly saliva are particularly lacking. Once acquired through an infected blood meal, the BSF parasites encounter a number of physical and immunological barriers in the gut [9], [10], [11], such as the peritrophic matrix [12] and a battery of host immune molecules, including reactive oxygens (ROS), antimicrobial peptides (AMPs), Peptidoglycan Recognition Proteins (PGRPs), tsetse EP Protein which restrict the establishment of successful infections [12], [13], [14], [15], [16], [17], [18], [19], [20]. In susceptible flies BSF parasites differentiate in the midgut into procyclic (PC) cells, which are characterized by an invariant surface coat made of procyclin proteins, reviewed in [21]. The PC form parasites migrate to the proventriculus organ in the anterior midgut where they differentiate into epimastigote (EPM) cells [1]. The EPM subsequently migrate to the SG where they express a different invariant coat made of a family of glycosylphosphatidyl inositol-anchored proteins, *Brucei* Alanine-Rich Proteins (BARPs) [22]. EPMs ultimately develop into the non-dividing free MC parasites that detach from the epithelium and are injected in saliva to the next vertebrate host during blood feeding [23].

Arthropod saliva contains important pharmacological agents that interfere with vertebrate host responses to enable successful blood feeding, such as suppression of vasoconstriction, platelet aggregation and coagulation [24], [25], but can also modulate host immune environment at the bite site to impact pathogen transmission outcome. Among the known tsetse saliva components are anti-hemostatic proteins [26], [27], [28], which include a potent anticoagulant thrombin inhibitor (TTI) [29], [30], and an anti-thrombotic apyrase (5′Nuclease) with dual inhibitory action that can bind to the fibrinogen receptor (GPIIb/IIIa) and inhibit ADP-induced platelet responses [31]. In addition, two abundant proteins (Tsal1 and Tsal2) have been described with DNA/RNA non-specific nucleic acid binding [26], [32], [33]. Another abundant protein, Tsetse Salivary Gland Growth Factor-1 (TSGF-1), has been shown to have putative anti-platelet aggregating activity [34]. In addition to proteins with anti-hemostatic functions, immunogenic allergens have also been described in tsetse saliva, including Tsetse Antigen5 (TAg5) [32], [35], which belongs to the family of Cysteine-Rich Secretory Proteins and Pathogenesis-Related 1 Proteins found in insects [36]. TAg5 has been shown to sensitize mice and trigger acute hypersensitivity reactions through induction of IgE and activation of mast cells/basophils to release vasoactive mediators [35]. Additional components of tsetse saliva are hypothetical proteins with unknown functions, glycolipids, calcium ions, amino acids, inositol, glycoproteins, sugars and phospholipids [37], [38], [39].

Infection with both trypanosomes and an entomopathogenic virus (*Glossina pallidipes* Salivary Gland Hypertrophy Virus, GpSGHV) have been shown to modulate SG gene expression and saliva composition, presumably to either enhance pathogen colonization in SG, or to increase pathogen transmission and survival at the vertebrate bite site [40], [41]. Several tsetse saliva proteins have been shown to be reduced in parasitized SG, including TTI [29], [30], TSGF-1 and TSGF-2 [34], Tsal1 and Tsal2 [32] and TAg5 [32], [41]. Reduction in anti-hemostatic factors in saliva could reduce the fly feeding performance, and result in an increase in the number of bites the fly has to take to be fully engorged [40]. The increased host-biting frequency in turn will promote parasite transmission to a greater number of mammalian hosts. Additionally, saliva from parasitized flies has been shown to decrease the expression levels of host proteins at the intradermal injection site, such as interleukin IL-6 and IL-12, as well as tumor necrosis factor (TNF), which in turn can skew the host immune responses to favor parasite survival [42].

In the present work, we used an RNA-seq approach to compare the transcriptome of the SG from uninfected and *T. b. brucei* infected *G. m. morsitans*, for which the whole genome sequence is now available [43]. In addition to the host SG transcriptome, we also identified the trypanosome-specific transcriptome from infected SG, and compared this to the BSF transcriptome we obtained from the same strain of parasites replicating in murine blood. We report on the differentially regulated SG genes, whose products may be critical for parasite differentiation in the SG or survival in tsetse, or for parasite establishment processes in the micro-environment of the vertebrate host bite-site. In addition, we describe the stage-specific transcriptomes of the parasite forms that reside in the salivary glands (referred to as SG-parasites) and mouse blood (BSF) that provide insights into parasite adaptations to the varying nutritional ecology and immune responses of the two different hosts.

## Results

### RNA-seq data from uninfected and parasitized SG reveals genes preferentially expressed in SG and parasite-induced changes

**Figure 1:**
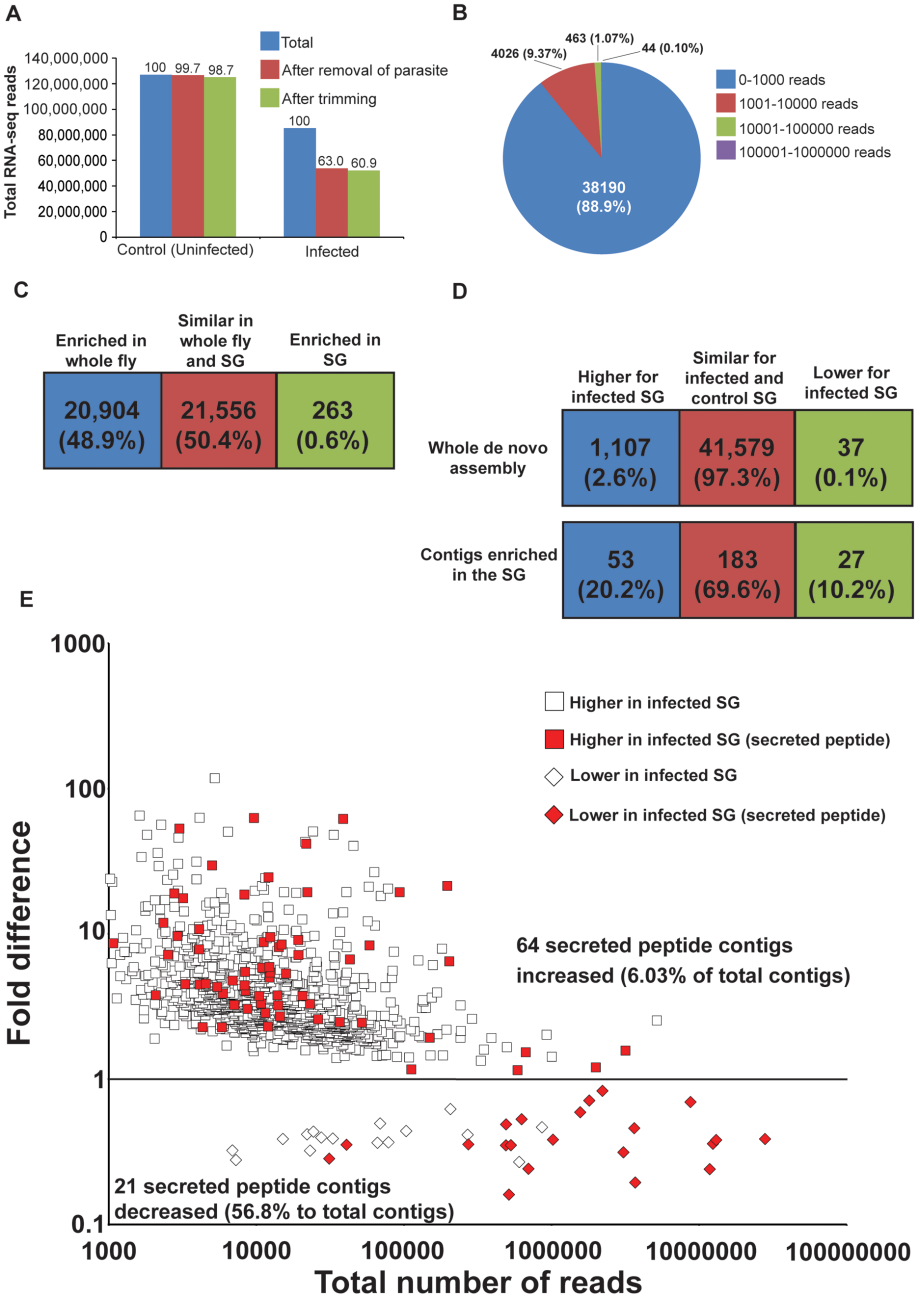
Overview of salivary gland transcriptome analysis. A. Number of RNA-seq reads after quality control and removal of parasite reads. B. Reads per contigs for control salivary gland. C. Contigs enriched in salivary glands (SG-enriched data set) in relation to whole female fly transcriptome data. D. Contigs with higher or lower expression following parasite infection in the salivary glands. E. The abundance of reads and fold difference of expression per contig are shown from parasite infected (shown in red) and from normal (shown in white) salivary glands. Those denoted below the line indicate reduced expression while those above indicate increased expression. The red dots below the line indicate the 21 secreted peptide contigs that correspond to 56.8% of total contigs.

To expand information on salivarian trypanosomes and on SG-trypanosome interactions, we performed two RNA-seq projects from normal SG (referred to as SG) and infected SG (referred to as infected-SG). Bioinformatics analysis of these two projects produced 120 million and 80 million reads from the SG) and infected-SG datasets, respectively (Figure 1A). Removal of low quality reads after trimming resulted in removal of less than 3% of the reads in each library. Elimination of parasite specific reads from the infected-SG library further reduced the number of reads by 37% (Figure 1A). Mapping of SG Illumina reads to a previous *de novo* assembly of female whole body reads [44] indicated that most of the contigs (88.9%) have less than 100 mapped reads, while 507 contigs were represented by over 10,000 mapped reads (Figure 1B). We next compared the SG library to the whole body female transcriptome data from Benoit et al. [44] to identify transcripts that were preferentially enriched in the SG under normal physiological conditions. This analysis indicated that over 20,000 contigs (nearly 99.4%) had similar levels of expression between whole flies and salivary glands (Figure 1c). Only 263 contigs (0.6%) were enriched in the salivary glands (Figure 1C, Table S1). This salivary gland enriched set (denoted as SG-enriched) of 263 contigs, was utilized throughout our analyses in addition to the complete contig library to examine changes that occur in the salivary glands during parasite infections.

In order to validate the transcriptome data, we selected *β-tubulin*, *28S ribosomal RNA*, and *GAPDH* as candidate genes to be used as reference for validation. Expression level of each gene was evaluated between infected and uninfected SG samples by qRT-PCR analysis. We found the least variable gene to be *GAPDH*, with a stability value of 0.535 based on Normfinder analysis [45], while *β-tubulin* was found to be the most variable due to its increased expression in infected SG. For transcriptome validation, we included genes previously shown to be differentially expressed upon infection [40], as well as several additional genes selected from the current transcriptome analysis (Text S1). These results collectively validated the data transcriptome data obtained using the RNA-seq analysis between control and infected SG.

Trypanosome infection resulted in increased expression levels for 1,107 (2.6%) transcripts when the entire contig library was analyzed, and for 144 (75%) transcripts when the SG-enriched contigs were considered (Tables S1 and S2). For transcripts that were reduced in expression during infection, only 37 (0.1%) were identified from the complete contig library and only 27 (less than 10%) from the SG-enriched dataset (Figure 1D). In general, the majority of the contigs reduced in expression upon parasite infection were expressed at high levels (high total number of reads), and the majority of these contigs encoded predicted secretory peptides (Figure 1E). Thus, transcripts that were reduced in parasitized SG comprised over 57% of the total reads in the SG-enriched library.

### Gene ontology (GO) analysis of SG transcriptome during trypanosome infection

**Figure 2:**
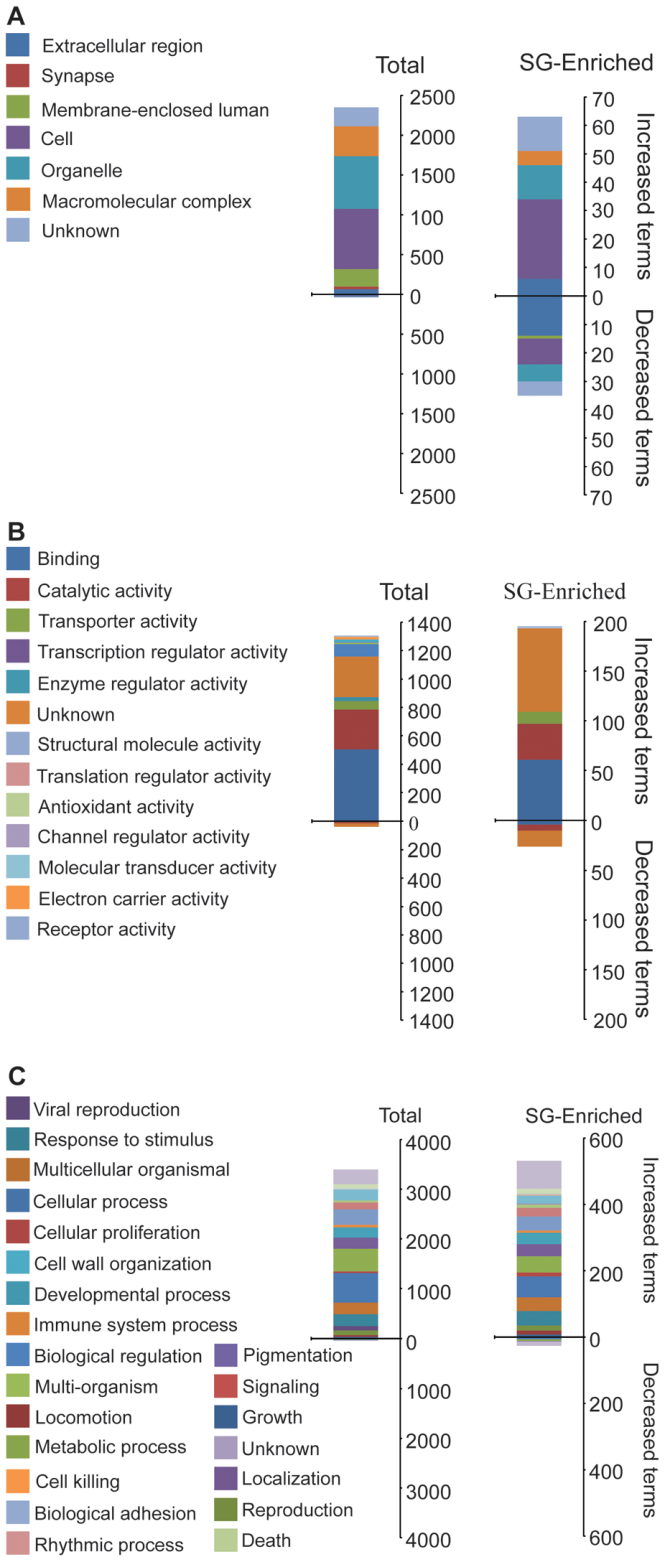
Functional classification based on gene ontology (GO) analysis of differentially expressed contigs in uninfected and parasite infected flies. A. Cell component. B. Molecular function. C. Biological Process.

GO analysis for cell component, molecular function and biological processes were performed for genes that displayed increased and decreased transcription levels (Figure 2). With respect to cell component analysis, there was enrichment of SG-enriched contigs involved in cell structure and macromolecular complexes (Figure 2A). A significant decrease was noted in SG-enriched genes that are components of the extracellular matrix (i.e. secreted proteins; Figure 2A). For the molecular function category, contigs involved in binding, catalytic activity and of unknown function were enriched in the SG-enriched dataset, but most of the contigs that were decreased were of unknown function (Figure 2B). With respect to the biological processes, there were no specific categories that were either enriched or decreased (Figure 2C).

**Figure 3:**
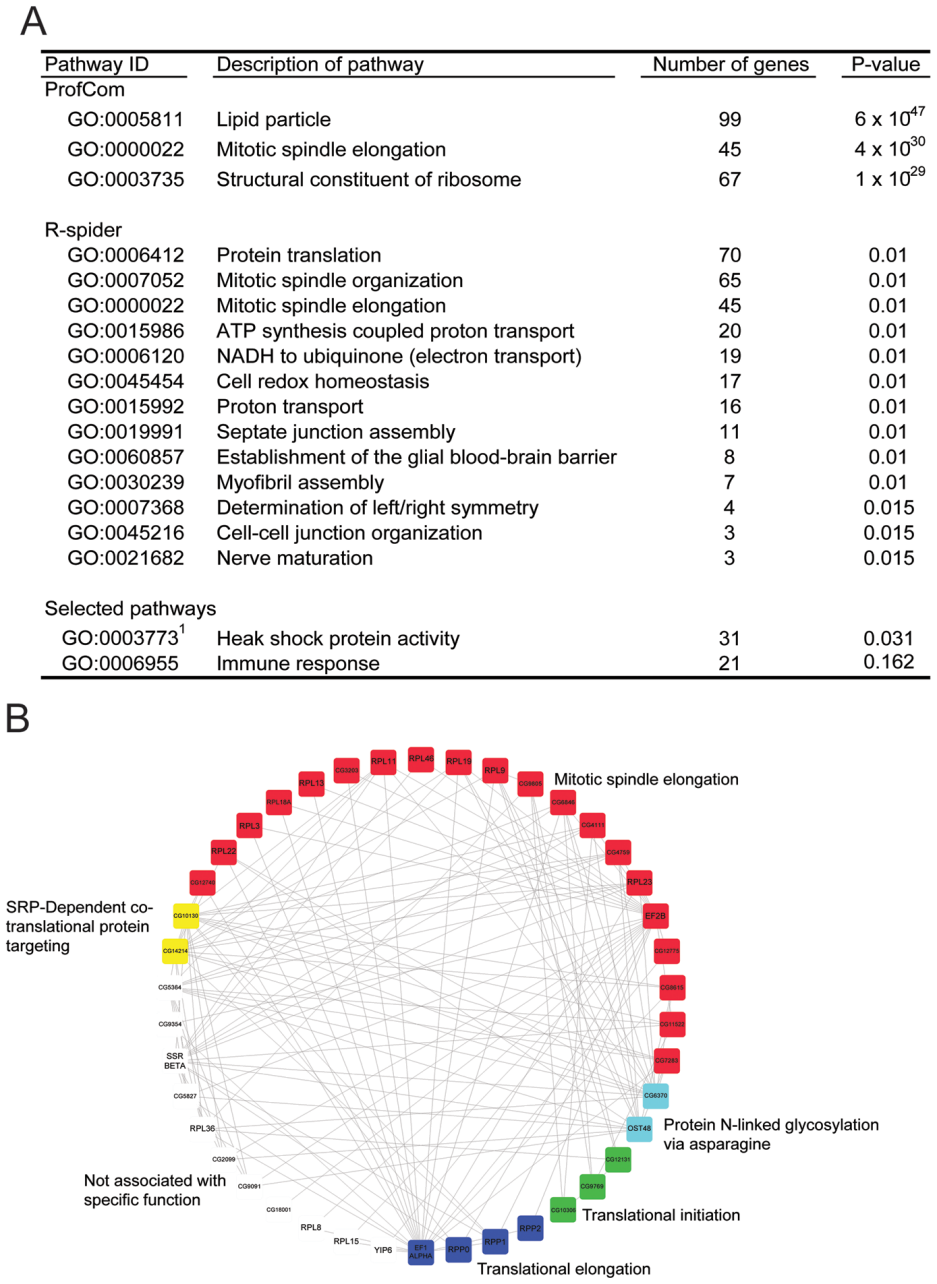
Gene ontology (GO) enrichment analysis of contigs increased during trypanosome infection in the salivary glands. A. Pathways enriched determined through ProfCom [98], R-spider [46] and selected pathways (P<0.05). B. Enriched networks identified allowing for no missing genes based on *Drosophila* homologs determined through R-spider [46].

We used *Drosophila* genes as proxy for tsetse genes to identify potential interactions between specific pathways using the R-spider program [46]. Multiple pathways were identified as enriched during parasite infection, including those involved in cell division (mitotic spindle organization and elongation), protein translation, ion transport (ATP synthesis couple proton transport and NADH to ubiquinone), cell redox homeostasis and heat shock proteins (Figure 3A). Using a model that allowed for no missing genes, we identified a network that consisted of 42 genes with increased expression in infected SG (Figure 3B). The putative gene products of this network are primarily associated with protein synthesis and mitotic spindle development, suggesting that parasite infections in salivary glands result in an increase in host cell division due to tissue damage from infections.

### Transcript levels of abundant secreted proteins are reduced in parasitized SG

**Figure 4:**
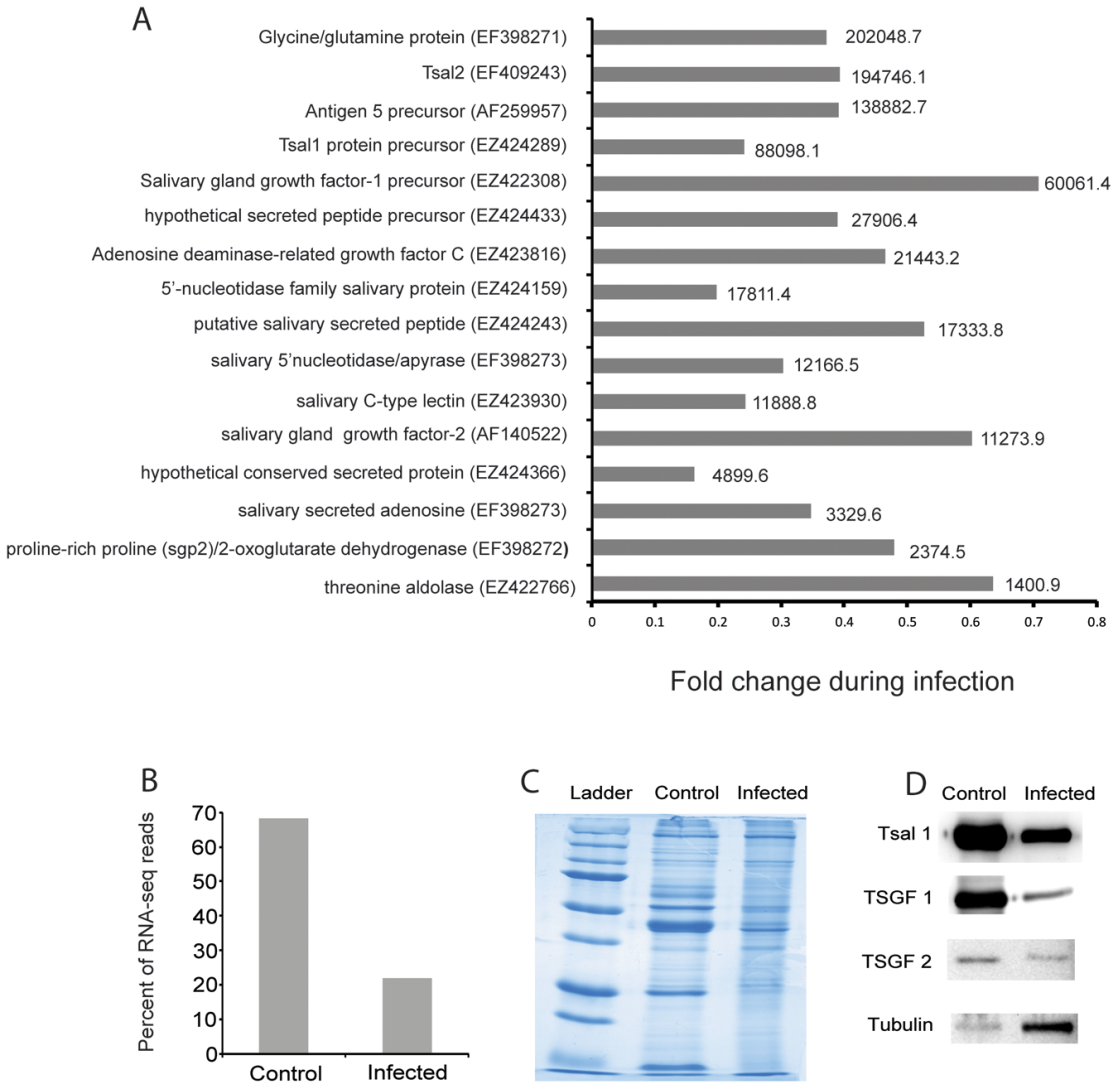
Specific salivary gland contigs and proteins suppressed during trypanosome infection. A. Fold change during salivary gland infection based on RNA-seq analysis from SG-enriched dataset that have a combined infected and control RPKM value of over 1000 from Table S1. The combined infected and control RPKM value is shown next to each bar. B. Percent of RNA-seq reads represented by the contigs with decreased expression during trypanosome infection. C. Total protein profile analyzed by SDS-PAGE analysis from one pair of infected and control salivary glands, one representative sample is shown. D. Western blot analysis of Tsal1, TSGF-1, TSGF-2 as well as Tubulin from control and infected SG.

We next analyzed the SG genes that were reduced in expression upon parasite infections (Figure 4A; Table S1, Table S2). The expression levels for several of the contigs (*tag5*, *tsal1-2*, *tsgf1-2*, *5′-nuc* and *sgp3*) identified as down-regulated by the RNA-seq analysis have also been previously noted to be decreased upon parasite infections (Figure 4A) [40]. One exception we noted was the major anticoagulant TTI, which unlike what has been reported in Van den Abbeele 2010 [40], did not reveal a significant reduction in gene expression in parasitized SG by RNA-seq analysis. In addition to the previously identified genes, we found that expression levels of other contigs classified as SG-enriched were also decreased in parasitized SG, including multiple secreted peptides with unknown functions and two C-type lectins [26]. We also identified a significant decrease in the expression levels of contigs encoding putative threonine aldolase, alkaline phosphatase and combined contigs that included SGP2 and 2-oxoglutarate dehydrogenase in infected-SG. The suppressed contigs represented over 20% of the total reads from the infected-SG library, and about 55–60% of the total reads in the SG enriched library (Figure 4B).

The observed drastic reduction in gene expression in parasite infected SG may account for the 3-fold decrease in protein levels that are reported in infected SG in comparison to normal SG [40]. We next analyzed protein levels in parasitized SG by SDS-PAGE analysis, and for several proteins for which we had antibodies by Western blot analysis (Figure 4C and 4D). SDS-PAGE analysis showed that some of the abundant proteins were significantly reduced in trypanosome infected SG samples (Figure 4C). Western blot and densitometry analysis confirmed that the three most abundant saliva proteins, Tsal1, TSGF-1 and TSGF-2, were reduced in infected SG in comparison to normal SG by 4.3, 19.7 and 2.7 fold, respectively (Figure 4D). Interestingly, β-Tubulin level was also increased by over 10 fold in infected SG relative to normal SG controls (Figure 4D). Our transcriptome and qRT-PCR-based analysis have also indicated a similar level of increase for *β-tubulin* gene expression in infected SG (Table S1; Table S2). Collectively, the transcriptome and protein data suggest that parasite infections result in a drastic reduction in levels of the major SG-enriched proteins. This finding suggests that the composition of infected fly saliva is likely to be drastically different from the normal state given that most of the reduction in SG is associated with secreted proteins. Since tsetse saliva has been shown to modify the mammalian host responses, drastic reduction in saliva composition can likely influence infection outcomes with trypanosomes at the bite site.

### Immune-associated gene expression in parasite infected SG

Contigs directly involved in immunity, or which may have an underlying role in immunity, were determined by searching our entire contig-library for *Glossina* immune genes and immune related genes from *Drosophila*. For this analysis, we first combined *Glossina* genes known to be involved immunity and these were Blastn searched against our *Glossina* contigs. Second, we generated a *Drosophila* immunity gene set by combining genes that have a GO function associated with immunity and by including genes, which have been cited in studies involving *Drosophila* immunity [47], [48]. This analysis yielded over 200 genes that were subsequently tBLASTx searched against the *Glossina* contigs. Following removal of contigs with an E-value higher than 10^−70^ for the *Glossina*-specific genes and 10^−30^ for the *Drosophila* genes along with those not significantly different between infected and control SG, 75 immune-associated contigs remained (Table S3). We observed that with the exception of the Nucleolar transcription factor 1-B, expression of all immune-related genes was increased in response to trypanosome infection. Among the genes identified were multiple contigs encoding serine protease inhibitors (*serpin4* and *serpin6*) that are increased 19–25 fold, respectively, *thiolester containing protein* variants (including *tep2*) that are increased 8–63 fold as well as multiple contigs associated with ubiquitination pathways and members of the GTPase family (Table 1). In addition, we found that abundance of two lectin transcripts are significantly reduced in parasitized SG (Table 1). Lectins can play important roles by recognizing pathogen associated molecular patterns and activating the lectin complement pathway by binding to carbohydrates expressed on parasite glycoproteins [49]. Reduction of lectin levels in the midgut in the initial stages of infection has been shown to increase the maturation of parasites [50]. It remains to be seen whether lectins can also interfere with the EPM establishment processes in the SG. The increased expression of TEPs in the tsetse SG suggests a host response that may involve the complement system as has been reported in mosquitoes [51] and ticks [52].

**Table 1:** Host immunity genes from tsetse differentially expressed in infected SG compared to uninfected SG.

GTPases		Fold change	Bonferroni	Control RPKM	Infected RPKM
ab-tri_asb-57940	GTPase Rab1/YPT1 small G protein superfamily	3.120301	9.8E-05	26.56479	83.19823
ab-tri_asb-12950	GTPase Rab2 small G protein superfamily	4.617021	0.015397	9.364569	43.45896
ab-tri_asb-7076	GTPase Ran/TC4/GSP1 mall G protein superfamily	4.05395	0	51.76676	210.8207
ab-tri_asb-4403	Rab30, isoform B	5.007407	3.1E-06	13.45945	67.75173
ab-tri_asb-14790	Rab protein 1	4.185714	9.49E-11	48.94724	205.5537
**Ubiquitination**					
ab-tri_asb-15967	Ubiquinol-cytochrome C reductase complex 11 kDa protein	2.594864	5.69E-11	69.95626	182.3178
ab-tri_asb-5799	ubiquitin activating enzyme 1	3.716578	0.000123	18.6399	69.63385
ab-tri_asb-35890	ubiquitin conjugating enzyme, isoform A	4.410405	1.31E-06	17.22152	76.45615
ab-tri_asb-55539	ubiquitin protein ligase	2.991597	0.002685	23.78252	71.3125
**Thiolester containing proteins (TEPs)**					
ab-tri_asb-23380	TEP2 protein	7.843434	1.14E-10	19.72284	155.5768
ab-tri_asb-57751	thiolester containing protein III	62.875	9.74E-09	0.817314	50.37243
**Serine protease inhibitors (Serpins)**					
ab-tri_asb-55758	serine protease inhibitor 6	24.55	0	7.951141	196.8012
ab-tri_asb-16075	serine protease inhibitor 4	18.68657	8.54E-11	6.66225	125.4431
**Lectins**					
ab-tri_asb-20511	salivary C-type lectin	−2.82524	0.000129	145.1698	51.60276
ab-tri_asb-55955	lectin	−2.39423	0.000933	173.9139	73.00219

### Comparative gene expression analysis between SG-parasites, including MC and BSF trypanosomes in vertebrate blood

**Figure 5:**
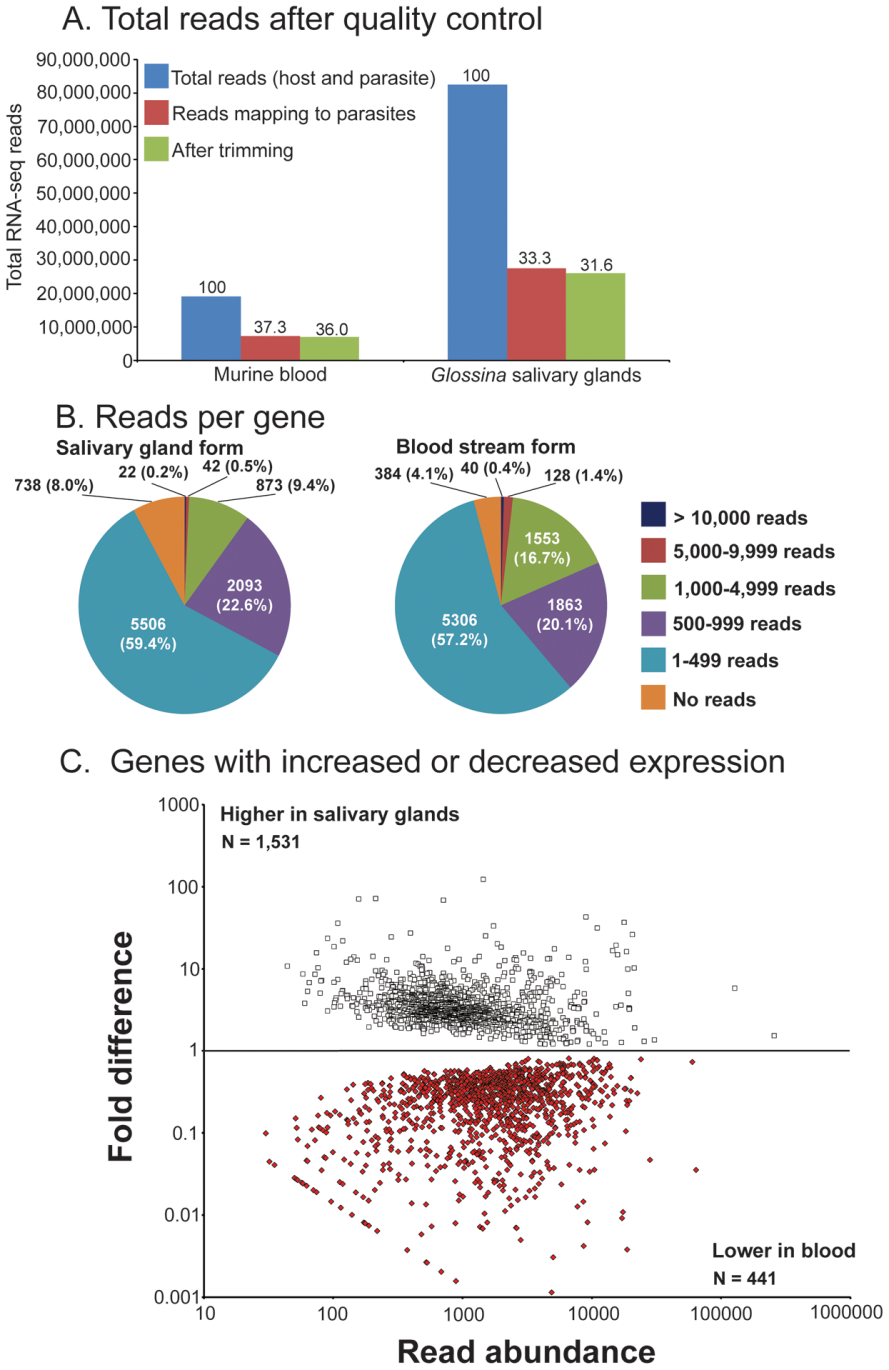
Overview of trypanosome transcriptome analysis. A. Number of reads mapping to the parasite protein coding genes from blood and parasite-infected salivary gland RNA-seq libraries. B. Reads per gene from parasite-infected blood RNA-seq library and parasite-infected salivary gland RNA-seq libraries. C. Genes expressed at significantly higher levels in parasite-infected blood and in salivary glands.

In addition to tsetse host genes, we recovered over 28 million parasite-specific reads, which corresponded to over 33% of total reads from the infected-SG RNA-seq library (Figure 5A). The parasite transcriptome in the infected-SG reads represent genes expressed in the various developmental forms of trypanosomes present in the SG, including the attached EPM, nascent-MC, partially coated pre-MC and mature non-dividing mammalian infective MC. We mined our data to understand the repertoire of genes that are expressed in these various SG developmental stages, which we collectively refer to as SG-parasites. In addition, we constructed and analyzed a third RNA-seq library from total RNA obtained from mice blood infected with the same parasite strain used for the SG analysis. From this sample, we obtained 7 million reads (37.3% of total), which represented transcripts specific for BSF parasites (Figure 5A). We compared the two transcriptome libraries to understand the differential expression of parasite genes in the two host environments. Mapping of reads to the parasites indicated that most of the genes have less than 1000 mapped reads (over 80% for parasites from blood and SG, Figure 5B). Few genes, less that 0.5%, had more than 10,000 mapped reads for both libraries (Figure 5B). There were over 1500 genes with significantly higher expression in the blood and over 400 with higher levels within the SG (Figure 5C). Validation of the transcriptome was accomplished through qPCR analysis of eight genes representing diverse functions (Text S2), and showed a high level of correlation (Pearson correlation = 0.985). Through this comparative analysis, we identified parasite specific genes that are differentially expressed in the salivary gland and host blood environment (Table S4 and Table S5).

Gene ontology enrichment through DAVID analysis [53], [54] revealed specific categories that were higher for either the SG-parasites or BSF stages (Table 2). In the SG-parasite stage, six GO categories were found enriched and included those involved in ion transport and protein synthesis. Examination of carbon source metabolism pathway [55], [56] from the SG-parasite data revealed that there are greater number of genes with higher expression level, whose putative products are associated with the tricarboxylic cycle, conversion of pyruvate to acetyl-CoA and metabolism of specific amino acids (alanine, threonine, glutamate and proline), confirming that amino acids represent the major source of energy for parasites in the tsetse host. In the BSF stage, there were five GO categories enriched, including glycolysis and metabolism of phosphorus (Table 2). In addition, pathways involved in folate metabolism, and amino acid biosynthesis had significantly more genes with higher expression level in parasites in SG than in BSF. We selected and describe below some of the most abundant known or hypothetical genes differentially expressed by parasites in the salivary gland and BSF stages.

**Table 2:** DAVID enrichment analysis for genes with BSF and SG-parasite expression profiles.

Higher in the blood	GO Number	Number of genes	Fold enrichment	Benjamini test
Nucleic acid transmembrane transporter	GO:0015932	9	4.074680924	0.032309994
Glycolysis	GO:0006096	8	5.768518519	0.03099419
Phosphorus metabolic process	GO:0006793	71	1.518999264	0.023106735
Cytoskeleton	GO:0005856	10	3.973333333	0.004969771
Protein kinase activity	GO:0004672	54	1.736749246	0.002219985

Higher in the salivary glands	GO Number	Number of genes	Fold enrichment	Benjamini test
tRNA aminoacylation for protein translation	GO:0006418	14	2.662046908	0.042085968
Ion transport	GO:0006811	31	1.964844147	0.041318542
ATP synthesis coupled proton transport	GO:0015986	13	3.009270418	0.029359114
Ribosomal subunit	GO:0033279	23	2.40390992	0.001334042
Translation	GO:0006412	92	1.88391012	1.85E-08
Structural molecule activity	GO:0005198	85	2.013623742	1.83E-09

Recent studies have applied *in silico* approaches to identify genes whose putative products are associated with cell surface components of trypanosomes and evaluated the development specific gene expression profile for some of these genes [57], [58]. These putative proteins typically carry signal peptide signals and GPI anchor motifs, or have predicted transmembrane domains associated with the product. We analyzed the differential expression of some of these putative proteins from the SG-parasite and BSF transcriptome data. These analyses recovered 62 genes that exhibit more than 4 fold differential expression in SG-parasites (Table 3). This dataset includes three amino acid transporter families, the family of BARP proteins and four cation transporters, all with high differential expression (Table 3). Additionally there is a hypothetical protein family represented by Tb927.7.360 that was expressed at 14 fold higher level in the SG stages. The five members of this putative family was previously shown to be expressed in the infective MC in tsetse saliva [57], and upon ectopic expression one member of this family was shown to localize to flagella in transgenic parasites [58]. We also observed that several different lineages of amino acid transporters were preferentially expressed in the SG-parasite stage; in particular three genes (Tb927.3.590; Tb927.8.7610 and Tb927.4.7740) showed over 10 fold differential expression. In addition, we detected multiple genes whose products function in the folate metabolism suggesting the importance of folate uptake and metabolism for parasites during their development in the tsetse host. The putative pteridine transporter family and the folate transporter (ESAG 10) were expressed 20 and 6 fold higher by SG-parasites than BSF stage, respectively (Table 3).

**Table 3:** Specific genes increased in SG-parasites from infected salivary glands.

Putative Function	Gene ID	RPKM SG	RPKM Blood	Fold Change
				MCF/BSF
Hypothetical Secretory Protein Conserved∧	Tb927.10.10770	1794	48	37
BARB Family*∧	Tb09.244.2400; Tb09.244.2410; Tb09.244.2420; Tb09.244.2430; Tb09.244.2440; Tb09.244.2450; Tb09.244.2460; Tb09.244.2470; Tb09.244.2480; Tb09.244.2490; Tb09.244.2500; Tb09.244.2510; Tb09.244.2520; Tb09.244.2530	7688	247	31
Pteridine transporter, putative Family*∧	Tb927.1.2820; Tb927.1.2880	2700	136	20
Hypothetical Protein Conserved Family*∧	Tb927.7.360; Tb927.7.380; Tb927.7.400; Tb927.7.420; Tb927.7.440	1031	93	14
Adenosine transporter*∧	Tb927.3.590	315	23	14
Purine nucleoside transporter (TbNT9)*∧	Tb927.6.220	316	24	13
Amino acid transporter 1, putative (AAT4/8)*∧	Tb927.8.7610	1663	166	10
Amino acid transporter, putative (AAT3)*∧	Tb927.4.7740	1173	70	17
Cation transporter, putative family*∧	Tb11.01.0725; Tb11.01.0730; Tb11.01.0760; Tb11.01.0770	1049	99	11
Folate transporter putative, Expression site-associated gene 10 (ESAG10) protein*∧	Tb927.8.3620	311	53	6
Hypothetical protein Family∧^@^	Tb927.2.4760; Tb927.2.4920; Tb927.2.5290; Tb927.2.5300; Tb927.2.5310; Tb927.2.5320; Tb927.2.5330; Tb927.2.5340; Tb927.2.5350; Tb927.2.5360	851	200	4
Hypothetical protein, conserved Family∧	Tb11.02.3760; Tb11.02.3770	4450	1286	4

Additionally, one abundant hypothetical secreted protein (Tb927.10.10770) was expressed at 37 fold higher level in the SG-parasite stages, and a family of hypothetical proteins (represented by Tb927.2.5300) with 10 members, all with predicted signal peptide and transmembrane domains, were expressed over 4–10 fold in SG-parasite stages in comparison to BSF cells. The *T. brucei* spp. specific Tb927.2.5300 family has been expanded in the *T. b. gambiense* 927 genome and has 24 members. In the BSF stages, 0.5% of the transcriptome was compromised of VSGs (including VSG 10.1 and a putative VSG Tb10.v4.0065) in addition to Expression Site Associated Genes (ESAGs) including ESAGs 1,2,3,9 and 11. In particular ESAG6/7, which encode the iron binding receptor Transferrin [59], were expressed over 200 fold higher level in the BSF than in the SG tissue (Table 4). In the SG-parasite library, less than 0.1% of the transcriptome, and only a single VSG (Tb927.5.3990) was expressed differentially. We noted three hypothetical putative proteins, Tb11.02.4450, Tb927.4.1910 and Tb11.47.0021, which had 856, 122 and 94 fold higher differential expression in the BSF stage, respectively (Table 4). None of these transcripts had associated signal peptide or transmembrane domains. Tb11.02.4450 was previously shown to have higher expression in the BSF than in PC culture forms [60]. We also detected adenosine transporters (TbNT3, 4 and 6), which showed 46–125 fold differential expression in the BSF stage. The nutritional ecology of the two hosts differs greatly. Reflective of metabolism difference, the BSF cells differentially express high levels of glucose transporters and glycerol kinase genes (Table 4).

**Table 4:** Specific genes increased in BSF parasites from mouse blood.

Putative Function	Gene ID	RPKM BSF	RPKM MCF	Fold Change
				BSF/MCF
Hypothetical Protein Conserved	Tb11.02.4450	428	0.5	856.0
Hypothetical Protein, Conserved	Tb927.4.1910	1237	54	22.9
Hypothetical Protein, Conserved	Tb11.47.0021	1132	12	94.3
Transferrin (ESAG6/7)*∧	Tb927.7.3250; Tb927.7.3260	2582	52	49.7
Hypothetical Protein, Conserved Zinc finger protein family member Putative (ZC3H11)	Tb927.5.810	6168	220	28.0
Hypothetical Protein, Conserved Zinc finger protein family member, putative (ZC3H20)	Tb927.7.2660	975	34	28.7
MSP-A*∧	Tb11.02.5610; Tb11.02.5630; Tb11.02.5640	528	16	33.0
RNA-binding protein, putative (RBP3)	Tb11.03.0550	481	6	76.0
RNA-binding protein, putative (DRBD12)	Tb927.8.6650	127	3	43.0
RNA-binding protein, putative (RBP9)	Tb11.01.3940	333	9	37.0
RNA-binding protein, putative (RBP26)	Tb927.7.3730	250	16	15.0
RNA-binding protein, putative (DRBD11)	Tb927.3.3940	235	22	11.0
cyclin-like F-box proteins	Tb927.1.4650; Tb927.1.4580	964	102	10.0
RNA binding protein (DRBD3)	Tb09.211.0560	861	107	8.0
RNA-binding protein UBP1 (UBP1)	Tb11.03.0620	814	146	6.0
Putative protein associated with differentiation Family^@^ (PAD1,3)	Tb927.7.5930; Tb927.7.5950	537	89	6.0
adenosine transporter 2, putative (TbNT3 and 4)	Tb927.2.6200; Tb927.2.6220	230	5	46.0
adenosine transporter 2, putative (TbNT6)	Tb927.2.6320	252	2	126.0
glucose transporter, putative	Tb927.10.8510; Tb927.10.8520; Tb927.10.8530	90	9	100.0
glycerol kinase, glycosomal (glk1)	Tb09.211.3540; Tb09.211.3560; Tb09.211.3570	517	3	172.3

Among the abundant and differentially regulated genes were members of the Major Facilitator Superfamily (MFS) Transporters known as Proteins Associated with Differentiation PAD. PAD1 and PAD3 were expressed at 6 and 7 fold higher level in BSF stages, respectively (Table 3). In contrast PAD 2, 4 and 6 were expressed at 20, 4 and 16 fold higher level in the SG-parasites, respectively (Table 3). Additionally we noted a large number of RNA binding proteins that were differentially regulated. In the BSF stage RBP3, DRBD12, RBP9 and RBP26 were abundant and expressed at 76, 43, 37 and 15 fold greater level than in the SG developmental forms (Table 4).

In summary, beyond metabolism differences, which reflect parasite adaptations to varying host nutritional environment, comparison of the BSF and SG-parasite transcriptomes display surface coat composition variations in the two different stages, which may reflect parasite adaptations to varying host immune biology. Our analysis also revealed many new and hypothetical proteins, which are abundant and differentially regulated in the two different stages, and which now await functional characterization.

## Discussion

In this study, we used a high throughput expression analysis to examine the tsetse-trypanosome interactions in the tsetse salivary gland. We analyzed parasite infection influences on its invertebrate host sialome by comparing the normal expression profile of tsetse SG with that of trypanosome infected glands. Our data reveal that parasite infections suppress the expression level of the most abundant secreted proteins in SG, thus likely changing the composition of, and the functional activities present in saliva. We also detected increased expression level of SG genes that are involved in immunity, stress tolerance and protein translation and cell division, suggesting that the parasite infections cause stress that necessitates cell growth and development in the glands. In addition to the host transcriptome, we also recovered gene expression information on the parasite from the SG developmental stages, and compared it with that of the transcriptome of the same parasite strain obtained from rodent blood. Comparison of the parasite transcriptome between the SG and mammalian stages revealed metabolic and surface coat protein shifts that may enable the adaptation of these two developmental forms to the varying immune and nutritional environment of their different hosts. In addition, we discovered many novel hypothetical and differentially regulated gene sets, for which no functional information is yet available. These discoveries now form the fundamental foundation necessary to advance the functional and mechanistic studies to understand host-parasite interactions at this critical developmental stage essential for disease transmission.

Using biochemical and molecular approaches, early studies on tsetse SG products identified five abundant secreted proteins [29], [30], [31], [32], [34], [35], [61], [62]. The influence of trypanosome infections on tsetse's saliva composition and functions was also reported and suggested a reduction for several of the abundant products upon parasite infections [40]. Subsequently a normalized cDNA library was generated and yielded over 20,000 ESTs from adult *G. m. morsitans* SG and considerably expanded the molecular knowledge on tsetse's physiology [26]. Combining the ESTs from the SG library with those from other normalized tissue libraries generated 2,509 novel putative proteins, 1,792 of which had at least one EST expressed in the SG and 59 of which were overrepresented in the SG library indicating high levels of expression. The EST-based study identified over 250 proteins uniquely associated with SG, of which about 20 were shown to be present in the saliva proteome. A proteomics based investigation on the saliva of a related tsetse species, *G. pallidipes*, identified 292 proteins that constituted the sialome of SG [41]. The comparative RNA-seq based analysis of normal and parasitized SG we report here expands upon these earlier discoveries, and provides insights into the pathways that dictate the tsetse- trypanosome cross-talk, which may enable successful parasite transmission and infection establishment in the mammalian host.

Our comparative analysis of the SG transcriptome to the whole body data identified 263 contigs that are preferentially expressed in the SG tissue, and included many of the previously reported major saliva proteins (Table S1). Our analysis on the infected SG data shows that the parasite infection causes an increase in transcript levels in 1,107 (2.6%) of genes that are not specific to the SG, but which are shared between different tissues represented by the whole body carcass. On the other hand, we detected that a much greater proportion (75%) of SG-enriched genes specific in function to the SG organ showed increased expression levels in infected glands, indicating that the infection also affects tissue specific functions. An example was *β-tubulin*, which was found to be higher at the RNA and protein level in infected SG, which suggests an ongoing structural reorganization in this organ. Among the genes that were increased in the SG-enriched dataset, we identified immunity, heat shock and stress related proteins. In tsetse stress related proteins have been identified [26], and shown to increase in expression upon SGHV infection [41]. The increased expression of these genes upon trypanosome infection in our analysis also highlights the heightened state of the host environment in the presence of the parasite. We also detected increased expression of cell growth related genes, which reflect tissue growth and maintenance in infected glands [63]. One additional group of genes related to cell and tissue growth or maintenance that was found highly expressed in the infected-SG dataset involves mitotic spindle formation during mitotic cell division [64], [65], [66]. High levels of infection with SGHV has been known to result in hypertrophied salivary glands [67], but the effect of trypanosome infections on the ultrastructure of the SG has not been documented extensively.

Our results showed that the majority of the SG transcriptome that was down-regulated in the presence of trypanosomes, encodes proteins secreted into the saliva. A reduction in levels of the abundant saliva proteins (TAg5, Tsal1, Tsal2, TSGF-1 and TSGF-2) upon trypanosome infection were also reported by Van Den Abbeele et al [40]. Infection of SG with high levels of SGHV pathogen was also found to reduce the abundance of the secreted saliva proteins by Kariithi et al. [41]. Interestingly, the major tsetse anticoagulant TTI did not reveal a significant reduction in gene expression in parasitized SG in our study, unlike the previously reported finding [40]. Our qPCR based expression data however showed high variability in *TTI* levels in individual infected flies (data not shown), suggesting that multiple factors may influence TTI levels, such as parasite infection densities as well as host digestive processes at the time of analysis.

It is possible that reduction of specific saliva proteins may also impact the EPM parasite attachment to the SG epithelia, which is necessary for the parasite to differentiate to mammalian infectious MC stages. Alternatively, given that the saliva proteins make up the major metabolic investment in this tissue, the drastic reduction in their levels may reflect an ongoing nutritional competition between the host and pathogen. It has been shown that in the triatomine bug *Rhodnius prolixus* infected with *Trypanosoma rangeli* (a SG invasive parasite), the parasite glycoinositolphospholipids cause gene expression alterations in the SG by inhibiting the SG protein tyrosine phosphatase activity [68]. More recently it was demonstrated that *T. rangeli* infection in the SG causes acidification and reduction of saliva proteins suggesting that the parasite may absorb un-specifically SG secreted protein for its survival [69]. Beyond enabling pathogen survival in the SG environment however, the reduced saliva proteins may also favor pathogen transmission to the mammalian host. This is because when tsetse flies probe the host skin, they create a hemorrhagic pool on which they feed and salivate, and in the process inoculate small amounts of saliva at the bite site. The array of pharmacologic activities with anti-thrombotic, anti-platelet aggregating and anti-coagulation activities are mainly designed to induce vasodilation and prevent blood clotting. A reduction in these activities has been shown to prolong the feeding time of tsetse, and increase the number of blood meals the fly has to take to reach engorgement [40]. This would in turn result in an increase in the transmission of the pathogen to a greater number of mammalian hosts. In the case of *Aedes* mosquitoes, infection with *Plasmodium* sporozoites caused an increase in the probing phase caused by reduced apyrase activity [70]. In triatomine insects infected with *T. rangeli*, it was observed that the number of bites on rabbit skin, the pace and amount of blood intake were all decreased due to a reduction of anticoagulant and apyrase activity in saliva [71].

Beyond enhancing direct transmission parameters, modification of the saliva composition may also impact disease transmission. This is because saliva has been shown to have immunosuppressive properties that may modify the host response at the bite site. Tsetse saliva has been shown to trigger acute hypersensitivity reaction, bias the immune response to Th2 and induce anti-vector antibodies [42]. Thus, a modified saliva composition can result in a more favorable immune environment at the bite site to enable trypanosome survival and/or differentiation from the MF to BSF forms in the mammalian host [42]. The tripartite relationships between vector saliva, pathogen and mammalian immune system are a newly expanding frontier with fundamental and applied implications for disease control. Our findings provide a first global insight into the many molecules involved in this dialogue, which can now be followed through functional investigations.

Knowledge on the molecular aspects of the tsetse-trypanosome interactions is limited in comparison to our knowledge on parasite's development in the mammalian host. Understanding tsetse proteins that contribute to parasite differentiation or replication in the midgut can lead to novel transmission blocking mammalian vaccines. Similarly discovering the MC surface proteome beyond the VSG coat can provide novel vaccine candidate antigens that can block transmission at the bite site. But molecular characterization of the final developmental stages in tsetse has been difficult since it has not been possible to culture the different forms of the MC cells *in vitro* to obtain enough biological samples for investigations. Only recently it was discovered that overexpression of a single RNA-binding protein, TbRBP6, in cultured noninfectious trypanosomes, leads to the generation of mammalian infective MC expressing the variant surface glycoproteins [72]. Thus, it will be now possible to obtain enough cells to expand into biochemical investigations with the MC specific proteins. In addition, advents of molecular methods, such as the one we applied here, which rely on small amounts of biological material now make investigations into MC biology feasible.

Our investigation on the SG-parasite transcriptome and its comparison to that of the BSF stages now provides the first global comparison between these two stages. This analysis has identified a large number of proteins that are to be likely involved in the post-transcriptional regulation of RNA processing mechanisms in the different stages of trypanosomes. Given that gene expression in trypanosomes largely relies on posttranscriptional control, the *T. brucei* genome encodes a large number of candidate RNA-binding proteins, which we found to be differentially regulated between the two developmental stages [73]. Recently two members of the Acetylation Lowers Binding Affinity family of proteins (ALBA3/4) with nucleic acid–binding ability have been shown to be colocalized with the DHH1 RNA-binding protein, and expressed in the parasite in the fly development stages. A role has been shown for ALBA3 in the EPM differentiation stages in the anterior midgut [74]. Our transcriptome analysis has uncovered differential expression of different members of the Major Facilitator Superfamily (MFS) Transporters, known as Proteins Associated with Differentiation PAD. The potential roles of PAD4, 6 and 8 in SG-parasite biology, and PAD1 and 3 in BSF stages in the mammalian host remain to be determined (Tables 3 and 4). A different member of the PAD family (PAD2) has been shown to be involved in the differentiation of BSF cells to procyclic insect stages early in the infection process in the fly midgut [75].

Recently, two *in silico* studies have mined the trypanosome genome databases to identify hypothetical surface proteins with GPI anchors [57], and the surface proteome of trypanosomes comprised of GPI anchor and transmembrane proteins [76]. Our SG-parasite expression analysis verify the abundant expression of proteins with known functions, such as the major EPM surface protein BARP, and cation, amino acid, pteridine and folate transporters. Nutrient transporters may be involved in the absorption of proteins or amino acids available in the SG lumen. In the case of *T. rangeli*, as well as other kinetoplastid organisms that lack heme biosynthesis pathway, parasites must acquire heme from their host [77]. Similarly, the BSF transcriptome verified the presence of antigenically variable VSGs, ESAGs, including the highly expressed iron receptor, Transferrin. Beyond the proteins with known functions, infected-SG data indicate high levels of expression for a hypothetical surface localized protein family with five members (represented by Tb927.7.360) with predicted GPI anchors, which is indicative of their surface localization. This gene family was shown to be expressed *in vivo* in the free MC stages in tsetse saliva [57]. In addition, ectopic expression of one member of this family (Tb927.7.360) with hemagglutinin (HA) epitope-tag at the N-terminal using an inducible expression vector co-localized the protein with the paraflagellar rod protein 1, consistent with its expression at or close to the flagellar membrane [58]. Interestingly, there is a single copy ortholog of the Tb927.7.360 family in the sequenced genome of *T. congolense* (TcIL3000.0.02370), which is also preferentially expressed in the EPM stage [78], indicating that this protein family may be involved in metacyclogenesis process.

Given that only a small number of parasites are transmitted in saliva to the mammalian host with each bite, the MC developmental stage represents a bottleneck that if effectively targeted could block the development of infections in the mammalian host. Towards this end, mice were immunized with radiation-attenuated parasites obtained from the mammalian blood 5-days post infection with fly challenge, before MC parasites switched to BSF, and shown to be protected against homologous MC challenge [79]. In addition, experiments where mice were challenged twice with *T. congolense* infected tsetse, with each challenge followed up by experimental cure, resulted in sterile immunity upon subsequent challenge [79]. However, immunity to cyclical challenge was short-lived and did not provide long-term protection to trypanosomes in these experiments [80]. Thus, knowledge on MCF specific proteins that interact with the host environment may advance the partial protection results obtained in these earlier studies. As such our analysis has uncovered a number of new candidate surface localized proteins, such as the Tb927.7.360 family described above, which can now be investigated for potential transmission blocking analysis. Similar studies on the sporozoite stages of *Plasmodium* spp. present in the mosquito SG have identified its major antigen CS protein as an important vaccine candidate to interfere with disease transmission [81], [82].

In conclusion, parasite infections in tsetse SG cause major modifications with structural and behavioral implications. For optimal transmission of the parasite in nature, it is important that SG infections do not reduce host fitness parameters, particularly longevity. This is because trypanosomes have a long extrinsic incubation period in tsetse, which is about 20–25 days before parasites acquired in the bloodmeal can mature to mammalian infective forms in the SG. In accordance, our prior studies had shown that parasite infection reduce tsetse's fecundity rather than longevity [83]. However, for optimal host longevity and parasite transmission to the mammalian host, it would be important that the blood feeding process is not compromised upon SG infections. Our transcriptome from infected SG provides molecular evidence of extensive cellular damage, as well as reduction in proteins with anti-hemostatic functions important in the blood feeding process. It is possible that the inflammatory responses elicited by SG in parasitized flies could influence trypanosome densities in the organ. It is also possible that the structural damage inflicted by parasite infections in the SG may be compensated by behavioral changes the infected tsetse host displays, such as more frequent blood meal acquisition. The frequent blood meals may serve for the nutritional needs of the tsetse host but at the same time result in the transmission of the parasite to many more hosts.

## Methods

### Biological samples

The colony of *G. m. morsitans* was established from puparia obtained in Zimbabwe in mid 1970s and has been maintained at Yale University insectary since 1993 under controlled temperature of 24±1°C with 50–55% relative humidity. The colony has been supplemented with puparia obtained from a similar colony maintained in Slovakia at multiple times over the years. Flies were fed on defibrinated bovine blood every 48 h using an artificial membrane system [84]. To obtain parasitized salivary glands, newly emerged (teneral) flies were fed on bovine blood supplemented with 2×10^6^ *T. b. brucei* (RUMP 503 strain) BSF parasites/ml. After a single infectious blood meal, flies were subsequently maintained on normal blood diets. For all experiments, salivary glands were dissected 72 h after the last blood meal when flies were 45 days old. Infection was confirmed by salivary gland dissections and microscopic examination of the parasites using a Zeiss Axiostar Plus light microscope.

The BSF *T. b. brucei* (RUMP 503 strain) were expanded in mice, and harvested from infected blood at peak parasitemia according to Yale IACUC protocol # 2011-07266. The BSF parasites were purified from blood cells using DEAE chromatography [85].

### RNA and protein preparations

The dissected salivary glands and the purified BSF parasites were transferred immediately to cold TRIzol Reagent (Ambion – Life Technologies, Carlsbad, CA) and kept under −80°C until total RNA was extracted using RNeasy Mini kit (QIAGEN, Germantown, MD) and stored at −80°C until cDNA library preparation. Total RNA was treated with turbo-DNase (Ambion) and absence of DNA was confirmed by PCR assay using the housekeeping gene glyceraldehyde-3-phosphate dehydrogenase (*GAPDH*) primers specific for tsetse [18], or *T. brucei* parasites (Table S6). For protein extraction, salivary glands were dissected, infection status confirmed as described above, immediately transferred to cold PBS buffer, added SDS-PAGE sample buffer, heated at 100°C for 10 minutes and stored at −20°C until used for Western Blot procedures.

### Salivary gland and BSF trypanosome cDNA library preparation and sequencing

Salivary gland cDNA libraries were prepared using TruSeq RNA kit (Illumina, Hayward, CA) according to manufacturer's protocol, with 1 µg of total RNA obtained from a pool of 30 pairs of uninfected or parasitized salivary glands, respectively. First strand cDNA was synthesized using random hexamers. Each uninfected or infected library was barcoded for Illumina HiSeq 2000 sequencing (paired-end 75 bp) at the Yale Centre for Genome Analysis (YCGA, New Haven, CT). BSF *T. b. brucei* 5′-end enriched library was generated by Illumina next-generation sequencing following published methods [72]. The Sequence Read Archive numbers at NCBI are: SG-control = SRR965340; SG-infected = SRR965341; mouse-blood infected = SRR965342.

### Bioinformatic analysis of tsetse salivary gland dataset

FASTQC analysis was performed on the RNA-seq datasets to determine read quality. Previously we have generated a whole body transcriptome of female flies and utilized this dataset to generate an annotated *de novo* contig library [86] using Abyss [87], [88] followed by assembly with Trinity [89], [90]. Parasite sequences were removed by mapping the Illumina reads to the *T. b. brucei* genome (strain 927; www.tritrypdb.org) prior to analysis. Transcript expression levels were analyzed using CLC Genomics Workbench (CLC bio, Cambridge, MA). RNA-seq datasets from infected and uninfected flies were mapped to the previous contig library with an algorithm allowing only two mismatches per read with at least 80% of the read matching the contig at 95% and a maximum of 10 hits per read. In addition, we conducted a secondary analysis comparing uninfected salivary glands and whole body RNA-seq databases [86] to determine contigs that were enriched in the salivary glands. Reads Per Kilobase per Million (RPKM) was utilized as a proxy of gene expression [91]. Relative number of reads for each contig in relation to the total read counts for each RNA-seq dataset was established to calculate P-value using Z-test following Bonferroni analysis [92]. Relative fold differences of contigs between infected and uninfected flies were determined as a ratio of the RPKM values and normalized based on the number of reads from each library. Blast2GO was utilized to determine the gene ontology (GO) terms for each contig that was differentially expressed between infected and uninfected datasets [93]. Enriched GO terms were determined using Fisher's Exact Test [93]. Pathways that were enriched in the control and infected samples were determined utilizing *Drosophila melanogaster* genes as a proxy in R spider network analysis (www.bioprofiling.de) [46]. R spider organizes gene products into pathways based on the Reactome signaling and KEGG metabolic networks to determine if interacting networks are over-represented. Immune-specific and associated genes for *D. melanogaster* were acquired from Flybase [48] through combining those with a GO biological function of immune response (GO:0006955) and genes recovered utilizing a search of “immune” along with adding known tsetse immune genes. A summarized overview of the transcriptome process is represented on Figure S1.

### Trypanosome transcriptome

We used two methods to analyze RNA-seq of parasites harvested from mice or present in infected salivary glands. In the first method, the RNA-seq libraries obtained from infected blood and salivary glands were directly mapped to the protein coding gene set predicted from the *T. brucei* genome (TREU927; tritrypdb.org) [76] using an algorithm that allowed for only two mismatches and a maximum of 10 hits per read with at least 80% of the read matching at 95% to the gene. Differences in expression levels and statistical analysis were conducted as described in the host transcriptome section. DAVID analysis was utilized to examine specific categories enriched with specific libraries [53], [54].

### Quantitative gene expression analysis for transcriptome validation

For tsetse and trypanosome specific gene expression analysis, we used five biological replicates, each containing one pair of either normal or parasitized salivary gland and three biological replicates of BSF. RNA was extracted as described and cDNA synthesis was carried using oligo-dT primers in a Superscript II reverse transcriptase reaction (Invitrogen – Life Technologies, Carlsbad, CA). Gene expression was evaluated by quantitative PCR (qPCR) with gene specific primers (Table S6) at 95°C for 5 min (1×), 95°C for 10 sec, 55°C for 10 sec, 72°C for 30 sec (45×) for tsetse and parasite genes. Gene expression levels were analyzed with CFX Manager software version 3.1 (Bio-Rad) and normalized to tsetse or parasite *GAPDH* gene chosen based on stability analysis for reference genes [94]. For tsetse, fold change in gene expression was calculated using infected salivary gland levels compared to non-infected gland levels. The fold change in parasite gene expression was calculated using MCF levels compared to the BSF levels. The transcriptome validation was achieved by determining the Pearson correlation between fold change obtained from qPCR compared to the RNA-seq data.

### Western blot

Protein extracts were obtained from pools of 20 pairs of normal or parasitized salivary glands and the corresponding amount of one pair of salivary gland was separated by 10% SDS-PAGE [95] and transferred to nitrocellulose membranes in semi-dry system at room temperature. Membranes were blocked with 3% BSA in PBS supplemented with 0.1% Tween-20, washed three times with PBS-T and incubated overnight with primary antibodies for anti-β-tubulin (1∶10,000) [96], anti-Tsal1 (1∶10,000), anti-TSGF-1 (1∶20,000) and anti-TSGF-2 (1∶5,000) [34], respectively at the indicated dilutions. HRP-conjugated goat anti-rabbit IgG at 1∶10,000 dilution was used as secondary antibody. The protein levels on western blots were quantified by densitometry analysis on reactive bands using ImageJ software [97].

## Supporting Information

Figure S1 Flow diagram of RNA-seq analysis of host and parasite reads from tsetse salivary glands.
(TIF)

Table S1 Contigs with enriched expression in the salivary glands (SG) compared to whole females [35] used for RNA-seq analysis comparing control SG and SG infected with trypanosomes.
(XLSX)

Table S2 RNA-seq analysis comparing uninfected salivary glands and those infected with trypanosomes from complete contig library form Benoit et al. [35].
(XLSX)

Table S3 Immune-associated contigs with differential expression based on RNA-seq analysis comparing control salivary glands (SG) and SG infected with trypanosomes.
(XLSX)

Table S4 Trypanosomes genes with significantly higher and over 5-fold difference in expression in the salivary glands compared to parasites recovered from mouse blood.
(XLSX)

Table S5 Trypanosomes genes with significantly higher and over 5-fold difference in expression in the mouse blood compared to parasites recovered from salivary glands.
(XLS)

Table S6 Primers utilized for tsetse flies and trypanosomes.
(DOCX)

Text S1 Validation of tsetse RNA-seq results with qPCR.
(DOCX)

Text S2 Validation of trypanosome RNA-seq results with qPCR.
(DOCX)
