# Supplementary figures and images for: Insights into the Trypanosome-Host Interactions Revealed through Transcriptomic Analysis of Parasitized Tsetse Fly Salivary Glands
Source: PLoS Negl Trop Dis. 2014 Apr 24;8(4):e2649. doi: 10.1371/journal.pntd.0002649 (PMC3998935; doi:10.1371/journal.pntd.0002649)

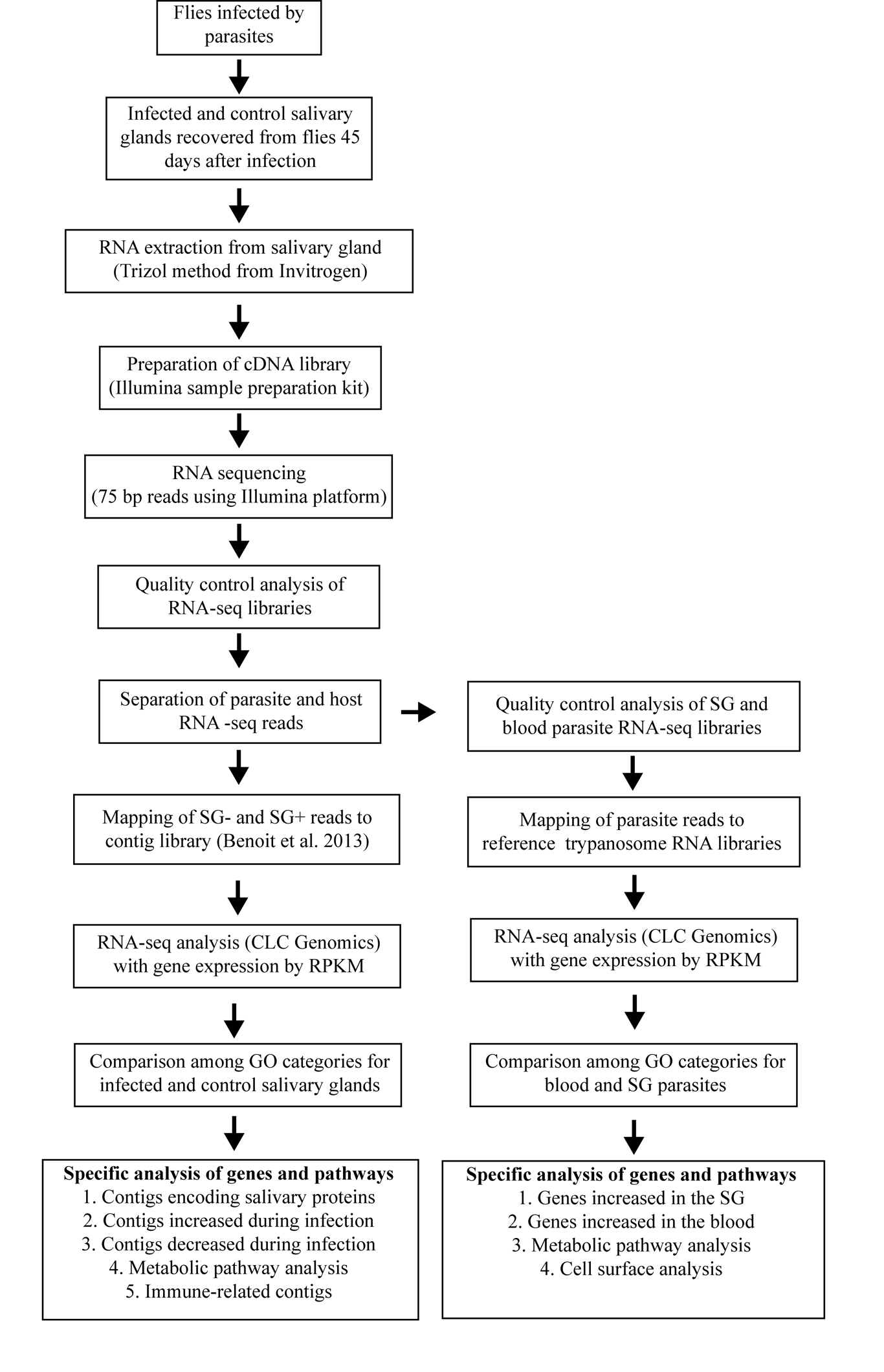

Supplement: Figure S1 — Flow diagram of RNA-seq analysis of host and parasite reads from tsetse salivary glands. (TIF) [file pntd.0002649.s001.tif]
