# Supplementary material for: Insights into the Trypanosome-Host Interactions Revealed through Transcriptomic Analysis of Parasitized Tsetse Fly Salivary Glands
Source: PLoS Negl Trop Dis. 2014 Apr 24;8(4):e2649. doi: 10.1371/journal.pntd.0002649 (PMC3998935; doi:10.1371/journal.pntd.0002649)
Supplement: Table S6 — Primers utilized for tsetse flies and trypanosomes. (DOCX) [file pntd.0002649.s007.docx]

| **Putative Tsetse Gene IDs** | **Gene Bank accession number** | **Forward primer** | **Reverse primer** |
| --- | --- | --- | --- |
| 28S rRNA | EF531135.1 | GTGATATAATGTGCCCAGTG | TGGTATCTGCATGAGTTGTT |
| Beta-tubulin | DQ377071.1 | CCATTCCCACGTCTTCACTT | GACCATGACGTGGATCACAG |
| DNA-RNA endonuclease (Tsal1) | EZ424289.1 | TGTAGTGGACAAGGAGAATG | TTGGCCTCTTCGCATTGATT |
| Gmm GAPDH | DQ016434.1 | CTGATTTCGTTGGTGATACT | CCAAATTCGTTGTCGTACCA |
| Hypothetical conserved protein B | EF398271.1 | GAGGTGACGGAAAGAATGCC | CCTCCACCTCCAAGGAATC |
| Hypothetical secreted peptide precursor | EZ424433.1 | GCTGTTATCCGTTGTTATTG | GTTACGCCATCTCCTGGTT |
| Iron Zinc transporter | EZ422920.1 | GGAATTTTGGGAGCTGTG | GTGAGCTTCGGGTAATATGTC |
| Salivary secreted protein | EZ423914.1 | TACCAACAACCGTTAATCCA | TTAATGGTTTCGCTTCGGT |
| Thrombin inhibitor (TTI) | AF054616.1 | ATCTGATAGTTGCCGCAC | AATTAAAGCCTTATGCCAGG |
|  |  |  |  |
| **Putative Trypanosome gene IDs** | **Tri-Tryp Gene ID** | **Forward primer** | **Reverse primer** |
| Amino acid transporter 1 | Tb927.8.7610 | TATTCGGATTCGCTGGTTC | AGTAACTGCCCAAATGGTA |
| Amino acid transporter 2 | Tb927.8.7740 | TATATTCCCAGCACTCTTCA | GTGTAATAAATGGACGTGCT |
| ATP-dependent phosphofructokinase | Tb927.3.3270 | CATGCGGCACAACAACTA | GTCGCGATTCCTGTTGAT |
| Cation transporter | Tb11.01.0725 | ATGCTGTTTGCACTACTCT | CAACATGAGGCAGAAAGC |
| Enolase | Tb927.10.2890 | ACCTTGTTGTTGCTCTTG | ACCTTGTTGTTGCTCTTG |
| Hypothetical protein 1 | Tb927.2.5530 | GAGAAGCGTCAGAAGGAA | GGATGGTGCCGATTTTAC |
| Hypothetical protein 2 | Tb927.2.5500 | AACCAACTACGGATCACT | CCATATAACTCTGTGTCCG |
| TbSGM2 | Tb927.7.400 | GTAAGTGCGGCATGTCTC | CATCTTGGCAACCTTCTCTC |
| Tryp GAPDH | Tb09.211.1370 | ATTCACGCTTTGGTTTGACC | GCATCCGCGTCATTCATAA |
