# Supplementary material for: Insights into the Trypanosome-Host Interactions Revealed through Transcriptomic Analysis of Parasitized Tsetse Fly Salivary Glands
Source: PLoS Negl Trop Dis. 2014 Apr 24;8(4):e2649. doi: 10.1371/journal.pntd.0002649 (PMC3998935; doi:10.1371/journal.pntd.0002649)

**Text S1**

**Validation of tsetse RNA-seq results with qPCR**. The RNA-seq expression values (log_2_ ratios) for 12 genes plotted against qPCR values (log_2_ ratios). The Pearson correlation coefficient (R=0.773) and goodness of fit (R^2^= 0.594) were high indicating correlation. This indicates the qPCR validates the tsetse RNA-seq data. * indicates data that were obtained from other publications in literature [31].

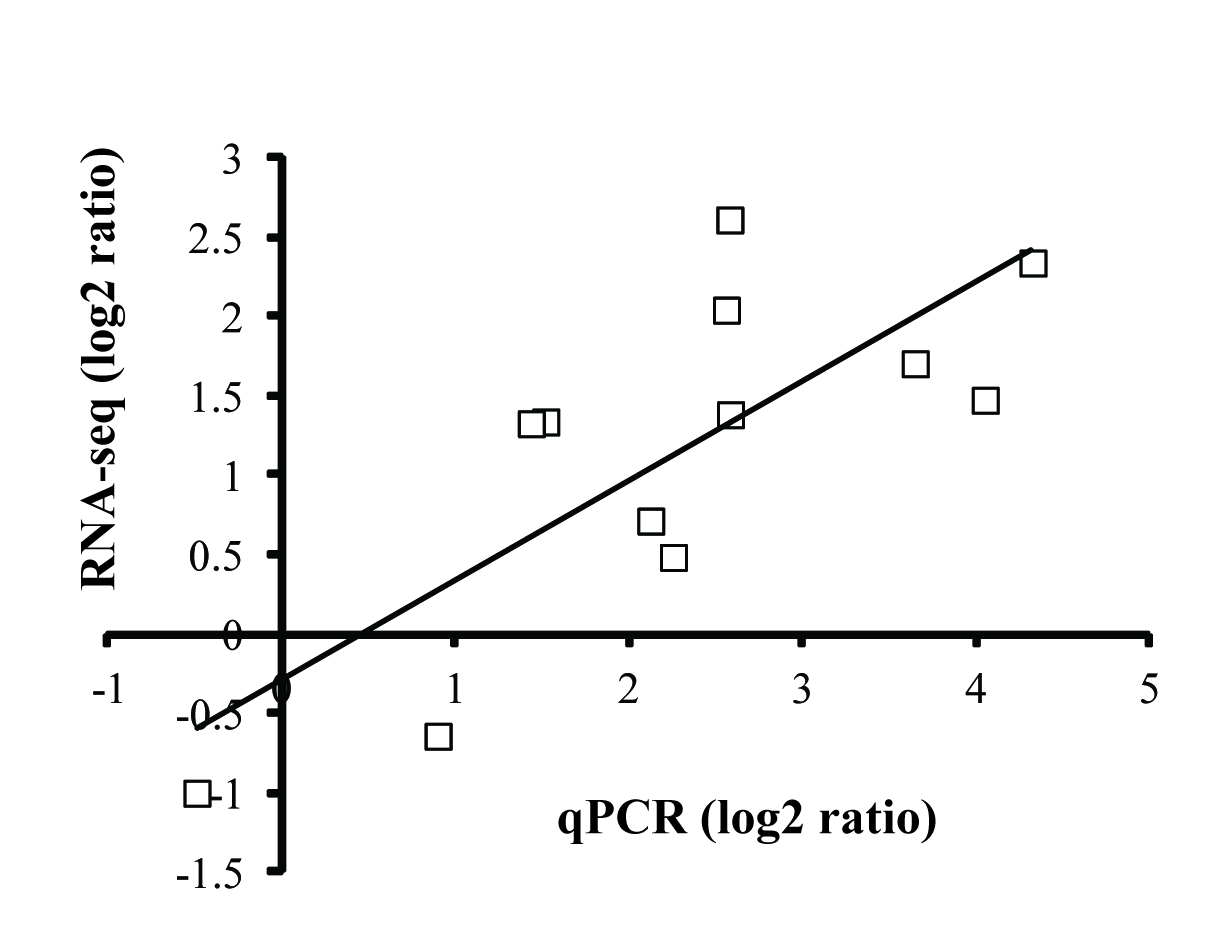

Supplement: Text S1 — Validation of tsetse RNA-seq results with qPCR. (DOCX) [file pntd.0002649.s008.docx]
