# Supplementary material for: Insights into the Trypanosome-Host Interactions Revealed through Transcriptomic Analysis of Parasitized Tsetse Fly Salivary Glands
Source: PLoS Negl Trop Dis. 2014 Apr 24;8(4):e2649. doi: 10.1371/journal.pntd.0002649 (PMC3998935; doi:10.1371/journal.pntd.0002649)
Supplement: Text S2 — Validation of trypanosome RNA-seq results with qPCR. (DOCX) [file pntd.0002649.s009.docx]

**Text S2**

**Validation of trypanosome RNA-seq results with qPCR**. The RNA-seq expression values (log_2_ ratios) for eight genes plotted against qPCR values (log_2_ ratios). The Pearson correlation coefficient (R=0.985) and goodness of fit (R^2^= 0.969) were high indicating a high degree of corelation. This indictes the qPCR validates the trypanosome RNA-seq data.

**
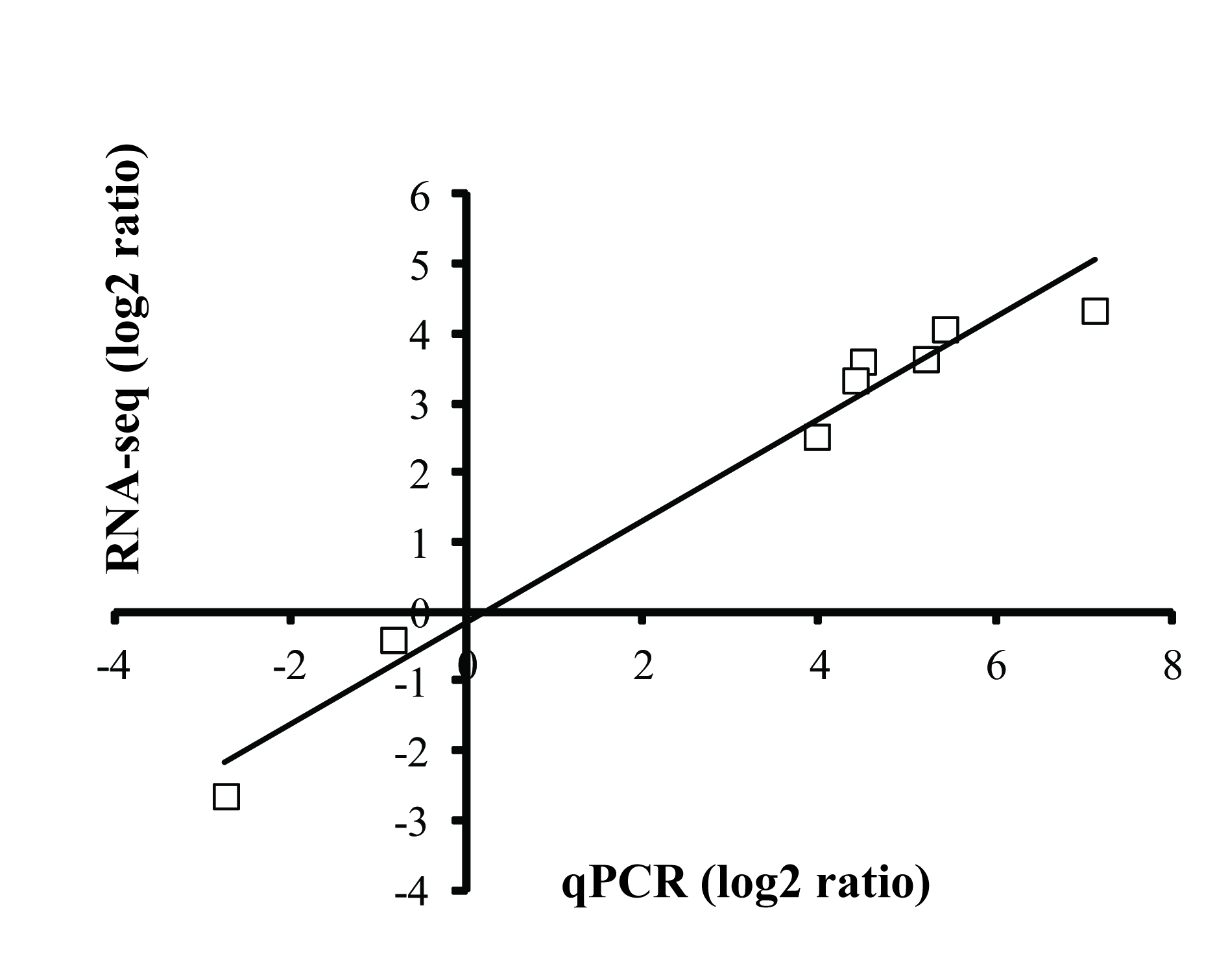
**
